# J. B. Flint & Co.

**Published:** 1892-02

**Authors:** 


J. B. FLINT & Co., New York, have in press and ready early in the current year, the following books:

A complete system of Gynæcology and Obstetrics, with 869 new illustrations based upon translations from the French of Pozzi, Auvard, and others, revised by Chas. Jewett, M. D., bound in leather or half morocco, $8.00.

Flint's Condensed Complete Encyclopædia of Medicine and Surgery, arranged upon a new system, and embodying the various methods of treatment employed by eminent practitioners. The most valuable and complete work of this nature ever published. The result of a year's labor of a large corps of writers. Leather or half morocco, two volumes, $8.00 per volume. The above works sold by subscription.

Also in press, ready March 1st, the Electro-Therapeutics of Gynaecology, by Augustin H. Goelet, M. D. Cloth bound, $2.50.

